# Semi-Interpenetrating Polymer Networks with Predefined Architecture for Metal Ion Fluorescence Monitoring

**DOI:** 10.3390/polym8120411

**Published:** 2016-11-29

**Authors:** Kyriakos Christodoulou, Epameinondas Leontidis, Mariliz Achilleos, Christiana Polydorou, Theodora Krasia-Christoforou

**Affiliations:** 1 Department of Mechanical and Manufacturing Engineering, University of Cyprus, 1678 Nicosia, Cyprus; christodoulou.c.kyriakos1@ucy.ac.cy (K.C.); achilleos.mariliz@ucy.ac.cy (M.A.); polydorou.christiana@ucy.ac.cy (C.P.); 2 Department of Chemistry, University of Cyprus, 1678 Nicosia, Cyprus; psleon@ucy.ac.cy

**Keywords:** semi-interpenetrating networks, fluorescent networks, anthracene, metal ions, fluorescence sensors

## Abstract

The development of new synthetic approaches for the preparation of efficient 3D luminescent chemosensors for transition metal ions receives considerable attention nowadays, owing to the key role of the latter as elements in biological systems and their harmful environmental effects when present in aquatic media. In this work, we describe an easy and versatile synthetic methodology that leads to the generation of nonconjugated 3D luminescent semi-interpenetrating amphiphilic networks (semi-IPN) with structure-defined characteristics. More precisely, the synthesis involves the encapsulation of well-defined poly(9-anthrylmethyl methacrylate) (pAnMMA) (hydrophobic, luminescent) linear polymer chains within a covalent poly(2-(dimethylamino)ethyl methacrylate) (pDMAEMA) hydrophilic polymer network, derived via the 1,2-*bis*-(2-iodoethoxy)ethane (BIEE)-induced crosslinking process of well-defined pDMAEMA linear chains. Characterization of their fluorescence properties demonstrated that these materials act as strong blue emitters when exposed to UV irradiation. This, combined with the presence of the metal-binding tertiary amino functionalities of the pDMAEMA segments, allowed for their applicability as sorbents and fluorescence chemosensors for transition metal ions (Fe^3+^, Cu^2+^) in solution via a chelation-enhanced fluorescence-quenching effect promoted within the semi-IPN network architecture. Ethylenediaminetetraacetic acid (EDTA)-induced metal ion desorption and thus material recyclability has been also demonstrated.

## 1. Introduction

The development of luminescent chemosensors for transition metal ions has attracted considerable attention in the last years given their high importance in biological systems [1], but also their harmful environmental effects when they are present in high concentrations in aquatic media [2,3]. To ensure high efficiency in a fluorescent metal-ion sensor, the light-emitting moiety should be covalently linked to a metal ion-binding group, and its fluorescence properties must be sensitive to the ion–ligand interaction [4,5].

Most literature examples focusing on 3D luminescent materials that are used as sensor platforms deal with metal–organic frameworks (MOFs) [6,7,8,9,10,11,12,13,14,15] and 3D coordination polymers [16,17,18,19,20]. Only a few research groups have been working on the synthesis of fluorescent semi-interpenetrating network (semi-IPN) architectures. The latter are 3D polymer structures consisting of secondary linear polymer chains that are interlaced—but not covalently bonded—with a primary polymer network [21,22,23,24]. In this limited number of existing reports, the fluorescent component is either a conjugated conductive polymer [25,26,27,28], fluorescent nanoparticles such as carbon nanodots and quantum dots (QDs) [29,30,31], or fluorescent dyes dispersed within or covalently linked to the polymer matrix [32,33,34,35]. However, the abovementioned materials have several disadvantages including: (a) low structural stability and robustness in the presence of chemical and physical impacts [36,37,38,39,40]; (b) possible leaching and relatively high probability of aggregation-induced fluorescence quenching of the physically entrapped organic dyes [41]; and (c) agglomeration phenomena of the fluorescent nanoparticles such as carbon nanodots resulting in inferior fluorescence properties due to self-quenching [42].

The necessity of developing functional organic polymer networks with controllable architectures have directed polymer chemists to explore new synthetic routes towards this purpose. Such materials are considered to be highly advantageous in comparison to their analogues prepared by a noncontrolled chemical crosslinking process, since the latter are usually highly inhomogeneous owing to the non-precise molar mass and broad molar mass distributions of the polymer segments constituting the networks. Consequently, polymer networks characterized by ill-defined architectures usually exhibit inferior mechanical and swelling properties, whereas their structural and compositional inhomogeneities restrict the structure-to-property correlation [43].

Although numerous examples on new synthetic approaches resulting in the generation of “model” or “quasi-model” covalent polymer networks have appeared so far, including “quasi-living” carbocationic polymerization [44,45], anionic polymerization [46,47], group transfer polymerization (GTP) [48,49,50], and controlled radical polymerization processes [51,52,53,54], there is only a limited number of publications discussing the synthesis of semi-IPN exhibiting structure-defined characteristics [55,56]. Very recently, our group has reported on the synthesis of such materials [57]. These consisted of well-defined hydrophilic and pH-responsive (poly(2-dimethylamino) ethyl methacrylate) (pDMAEMA) linear chains that were interconnected using 1,2-*bis*-(2-iodoethoxy)-ethane (BIEE) generating the pDMAEMA network, and well-defined hydrophobic poly(*n*-butyl methacrylate) (pBuMA) linear chains that were encapsulated within the network during the crosslinking process. From our studies, it has been demonstrated that the mechanical properties of these materials can be easily tuned by adjusting the content of the encapsulated hydrophobic linear chains, whereas their well-defined structural characteristics allowed for the prediction of their mechanical response via mathematical modeling.

Giving further credence to the BIEE-crosslinking approach as an alternative to synthetically demanding and multistep controlled polymerization processes, in the present study we report on the synthesis of 3D structure-defined emissive (fluorescent) amphiphilic semi-IPN, consisting of BIEE-crosslinked pDMAEMA segments and embedded hydrophobic and nonconjugated/fluorescent poly(9-anthrylmethyl methacrylate) (pAnMMA) linear chains both prepared by reversible addition–fragmentation chain transfer (RAFT)-controlled radical polymerization. Although in this case the coordinating active part (pDMAEMA) [58,59,60,61,62,63] is not covalently bound to the fluorescent active component (pAnMMA), the semi-IPN network architecture promotes the reinforcement of interactions between the pDMAEMA-complexed transition metal ions and the anthracene moieties of the interlaced pAnMMA chains, thus promoting chelation-enhanced fluorescence quenching. Moreover, interlacing of the pAnMMA chains within the nonfluorescent pDMAEMA network diminishes phase separation phenomena (and, consequently, self-quenching effects). Furthermore, the high molar mass and hydrophobicity of the macromolecular fluorescent pAnMMA prevents the leaching of the active component in polar and aqueous solvents.

These materials were further evaluated in the chemosensing of Fe^3+^ and Cu^2+^ transition metal ions. The former is a key element in biological and environmental systems, playing an essential role in oxygen uptake and metabolism and electron transfer processes [64]. Cu^2+^ ions, which are known to exhibit high affinity for N– and O–containing ligands, significantly contribute to the metal environmental pollution owing to their widespread industrial use. Even though the toxicity of Cu^2+^ ions is considerably lower compared to other heavy metal ions, very low Cu^2+^ concentrations are highly toxic to certain microorganisms [65].

Consequently, the presented versatile synthetic approach creates new prospects in the generation of nonconjugated 3D luminescent polymer-based sensors in which fluorescent moieties are combined with metal-chelating elements, characterized by structure-defined characteristics and tunable properties, with potential use in metal ion chemosensing.

## 2. Materials and Methods

### 2.1. Chemical Reagents

Poly(9-Anthrylmethyl methacrylate) (pAnMMA) (*M*_n_ = 27,900 g·mol^−1^; where *M*_n_: number average molar mass determined by size exclusion chromatography using poly(methyl methacrylate) (PMMA) calibration standards; polydispersity index, PDI: 1.3) and poly(2-(dimethylamino)ethyl methacrylate) (pDMAEMA) (*M*_n_: 19,000 g·mol^−1^, polydispersity index, PDI: 1.17), were in-house synthesized by RAFT-controlled radical polymerization according to our previous publications [5]. 1,2-*bis*-(2-iodothoxy)ethane (BIEE, Sigma-Aldrich, 96%, St. Louis, MO, USA), 9-anthracenemethanol (Sigma-Aldrich, 97%, St. Louis, MO, USA), methanol (MeOH, Sharlau, analytical grade, ACS reagent, Barcelona, Spain), and tetrahydrofuran (THF, Scharlau, HPLC grade, Barcelona, Spain) were used as received. FeCl_3_·6H_2_O (Sigma-Aldrich, ≥99%, St. Louis, MO, USA), Cu(CH_3_COO)_2_·H_2_O (Sigma-Aldrich, ≥99%, St. Louis, MO, USA) and ethylenediaminetetraacetic acid (EDTA) (Sharlau, 99%–101%, Barcelona, Spain) were used as received by the supplier.

### 2.2. Synthesis of Semi-Interpenetrating BIEE-Crosslinked pAnMMA/pDMAEMA Networks

By following a similar synthetic protocol as that described in a recent publication of our group [57], a semi-interpenetrating fluorescent polymer network of the type p(AnMMA)/pDMAEMA/BIEE was prepared via the encapsulation of pAnMMA (5 wt % in respect to the total polymer mass) within the BIEE-crosslinked pDMAEMA network. The experimental procedure is as follows. In a glass vial, pAnMMA (5 mg) was dissolved in THF (1.25 mL, 8% *w*/*v* solution concentration). To the solution, pDMAEMA (100 mg, 0.0052 mmol of macro-chain transfer agent, 0.64 mmol per DMAEMA unit) was added and the mixture was stirred rapidly until complete dissolution of pDMAEMA. To the solution, BIEE (58 μL, 118 mg, 0.32 mmol) was added using a micropipette. The resulting solution was stirred rapidly and was then left in a sealed vial at room temperature, under air and without stirring, until gelation was observed (7 days). The resulting network was then placed in excess methanol (100 mL) for one week to remove the sol fraction. The sol fraction (14%) was determined gravimetrically, and it was calculated from the ratio of the dried mass of the extractables to the theoretical mass of all components in the network (i.e., pAnMMA, pDMAEMA, and BIEE).

### 2.3. Swelling Behavior

The methanol-swollen network was cut into small pieces and their mass was determined gravimetrically before placing them in a vacuum oven to dry at ca. 25 °C for 24 h. The mass of the dry network pieces was then determined, and the degrees of swelling (DSs) in MeOH were calculated as the ratio of the swollen mass divided by the dry mass. Subsequently, the dried pieces were placed in deionized water for 2 weeks before determining the water-swollen network masses.

### 2.4. Fluorescent Characterization

The fluorescence emission spectrum of pre-swollen (in methanol) polymer network was recorded at the solid state by using a Jasco FP-6300 fluorescence spectrophotometer (Jasco Incorporated, Easton, MD, USA). The excitation wavelength was set at 370 nm, where AnMMA-containing polymers are known to exhibit maximum absorption [66]. Fluorescence microscopy was further used for visualizing the anthracene-containing fluorescent network at its swollen state (in methanol). The swollen network was placed in methanol-containing Petri dishes and examined under the Olympus fluorescence microscope (BX53 System Microscope, Olympus Corporation, Tokyo, Japan). The fluorescence intensity of the sample was determined by using the DAPI filter (U-FUNA, excitation: 358 nm, emission: 461 nm; Olympus, Tokyo, Japan). Other filters were used with different excitation and emission spectra, but no fluorescence was detected. Images were taken at 4× magnification and analyzed using the CellSens software. The same exposure time was used in all cases.

### 2.5. Fluorescence Monitoring of Cu^2+^ and Fe^3+^ Ions

Initially, Fe^3+^ and Cu^2+^ metal ion solutions of various concentrations were prepared by dissolving FeCl_3_·6H_2_O and Cu(CH_3_COO)_2_·H_2_O metal ion salts in methanol. More precisely, Fe^3+^ and Cu^2+^ methanol solutions were prepared, with concentrations in the range 5 × 10^−6^ to 5 × 10^−5^ M and 5 × 10^−5^ to 10^−3^ M, respectively.

The polymer network was cut in small pieces (m ~ 0.70 g), which were subsequently immersed in glass vials containing the metal ion (Fe^3+^ and Cu^2+^) solutions (5 mL) for 24 h. For comparison purposes, a control sample was also prepared upon immersing the polymer network in a glass vial containing pure methanol (5 mL). Afterwards, the pieces were washed twice with pure methanol and placed in new vials containing methanol (5 mL) prior to their characterization.

For comparison purposes, the fluorescence monitoring of Fe^3+^ and Cu^2+^ in methanol (concentration range 0.1–0.25 mM (Fe^3+^) and 0.1–1.0 mM (Cu^2+^)) was carried out by using the low molar mass 9-anthracenemethanol (1.25 mM) as the metal ion fluorescent chemosensor.

### 2.6. Desorption Studies—Polymer Network Regeneration

Initially, Cu^2+^-loaded polymer networks were prepared by immersing the methanol-swollen p(AnMMA)/pDMAEMA/BIEE networks in Cu^2+^ ion methanol solutions of two different Cu^2+^ concentrations (10^−3^ and 5 × 10^−3^ M) for 24 h followed by extensive washing to remove any unbound cations. For the regeneration process, the Cu^2+^-loaded polymer network pieces (m ~ 0.35 g) were immersed in aqueous EDTA solution (5 mL, 0.25 M) and they were visualized by fluorescence microscopy in real time.

## 3. Results and Discussion

### 3.1. Synthesis of Semi-Interpenetrating 3D Amphiphilic Fluorescent Networks

**Figure 1:**
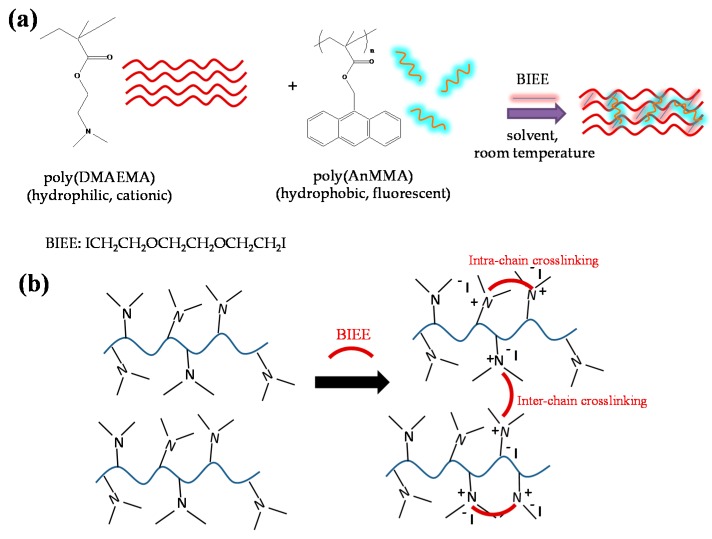
(**a**) Chemical structures of the linear poly(2-(dimethylamino)ethyl methacrylate) (pDMAEMA) and poly(9-anthrylmethyl methacrylate) (pAnMMA) homopolymers and of the 1,2-bis-(2-iodoethoxy)ethane (BIEE) crosslinking agent, and schematic of the one-step synthetic approach followed for the preparation of 3D luminescent semi-interpenetrating amphiphilic network (APN); (**b**) crosslinking reaction scheme presenting both, intra- and interchain crosslinking pathways that may occur between BIEE and –N(Me)_3_ pendant moieties of the poly(DMAEMA) linear chains.

The synthetic methodology followed for the preparation of structure-defined, BIEE-crosslinked fluorescent semi-IPN polymer networks was based on our recent publication [57]. Initially, pDMAEMA and fluorescent poly(AnMMA) linear homopolymers were prepared by RAFT-controlled radical polymerization. Unimodal polymers with controlled average molar mass (MWs) and relatively low PDIs were obtained in both cases [5,57]. The well-defined poly(DMAEMA) and poly(AnMMA) linear precursors were then dissolved in tetrahydrofuran (i.e., a good solvent for both homopolymers to ensure good intermixing at a molecular level) and employed as precursors for the generation of the BIEE/pDMAEMA/pAnMMA semi-IPN polymer networks, as schematically demonstrated in Figure 1.

The gelation process that was carried out at room temperature, under air and without mechanical stirring, was completed within 7 days. As already described in Section 2.2, the [BIEE]/[DMAEMA] molar ratio was fixed at 1:2, targeting a 100% degree of crosslinking, since 1 BIEE molecule is capable of linking together 2 tertiary amino functionalities. However, according to Armes and co-workers [67], intrachain crosslinking or reaction of one iodide group of the BIEE crosslinker may occur as a result of deviation from quantitative crosslinking. More precisely, quaternization of the PDMAEMA chains with BIEE may lead to either intermolecular crosslinking (branching) or intramolecular cyclization, as shown in Figure 1b. The sol fraction percentage (extractables) that was determined gravimetrically was 14%, which is in line with that reported in our previous study [57].

The networks’ swelling behavior is considered to be an important influencing parameter on their performance in metal ion uptake and sensing. The swelling behavior of the BIEE-crosslinked p(AnMMA)/pDMAEMA networks was investigated in both, water, and methanol. Based on the experimental data, the degree of swelling determined in water (19.1 ± 1.7) was comparable to that found in methanol (16.3 ± 0.8), suggesting that shifting from methanol to water would probably not lead to significant changes on the network’s sensing performance.

### 3.2. Fluorescence Properties

**Figure 2:**
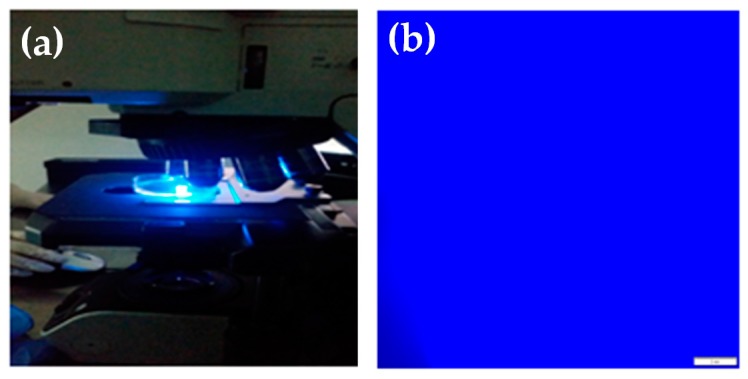
(**a**) Photograph of the BIEE/pDMAEMA/pAnMMA semi-interpenetrating network (semi-IPN) polymer network when exposed to UV irradiation, displaying strong blue emission; (**b**) corresponding fluorescence microscopy image. The scale bar in Figure 2b is 100 μm.

The fluorescence emission of the network pre-swollen in methanol was investigated by means of fluorescence spectroscopy and microscopy. As previously described in the experimental section, a BIEE/pDMAEMA/pAnMMA semi-IPN polymer network was synthesized, in which 5 wt % of the fluorescent pAnMMA component with respect to the total mass (polymers + crosslinking agent) was incorporated. As seen in Figure 2, the resulting network displayed strong blue emission (recorded at 461 nm) under 358 nm excitation wavelength. Moreover, the active fluorescent polymeric component was homogeneously distributed within the pDMAEMA polymer matrix, as verified by fluorescence microscopy.

**Figure 3:**
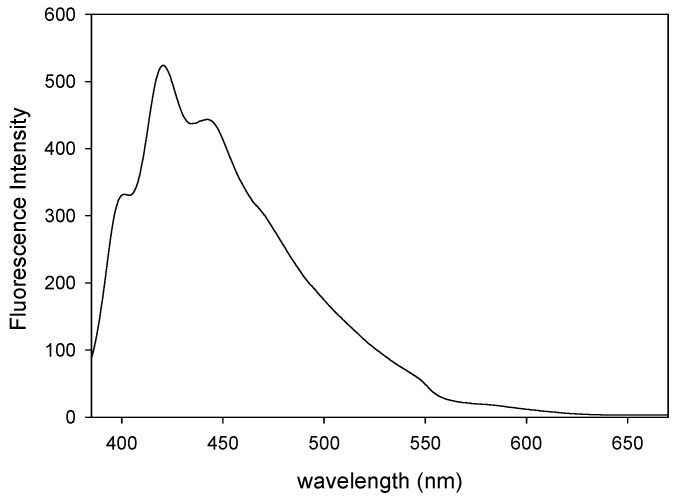
Fluorescence spectrum of the BIEE/pDMAEMA/pAnMMA semi-IPN polymer network pre-swollen in methanol. Excitation wavelength: 370 nm.

Fluorescence spectroscopy was used for recording the fluorescence emission spectrum of the methanol-swollen network. The vibronic structure of the network provided in Figure 3 resembled that of the anthracene fluorophore [66,68] and of the previously reported pAnMMA linear homopolymer analogue [5].

### 3.3. Fluorescence Monitoring of Cu^2+^ and Fe^3+^ Ions

The BIEE/pDMAEMA/pAnMMA semi-IPN polymer network system was examined regarding its ability to act as a macromolecular sensor for transition metal ions. More precisely, the study was focused on examining the chemosensing ability of such fluorescent 3D semi-IPN towards Fe^3+^ and Cu^2+^ ions dissolved in methanol. Both fluorescence microscopy and spectroscopy were used to monitor the qualitative and quantitative changes in the network’s fluorescence intensity when the latter was immersed in metal ion solutions of various concentrations prepared in methanol. The metal ion fluorescence monitoring studies were purposely performed in methanol, since this allowed for a direct comparison of the obtained fluorescence data with those acquired when using 9-anthracenemethanol (model compound) as a fluorophore (see Supplementary Materials), which is insoluble in water. Moreover, based on previous literature reports [69,70,71,72,73], the use of methanol or methanol/water mixtures of various volume ratios is typical in metal ion fluorescence monitoring studies, since methanol—being a protic and very polar solvent—exhibits properties similar to water. In one such example [73], the authors performed selective fluorescence quenching experiments by using an aziridine-based molecule possessing pendant anthracene units as a chemosensor, in both methanol and water as solvents, so as to determine the solvent effect on the quenching/enhancement mechanism. No significant differences were found in regards to the quenching of the fluorescence intensity in the presence of different metal ions when using water or methanol as solvents.

**Figure 4:**
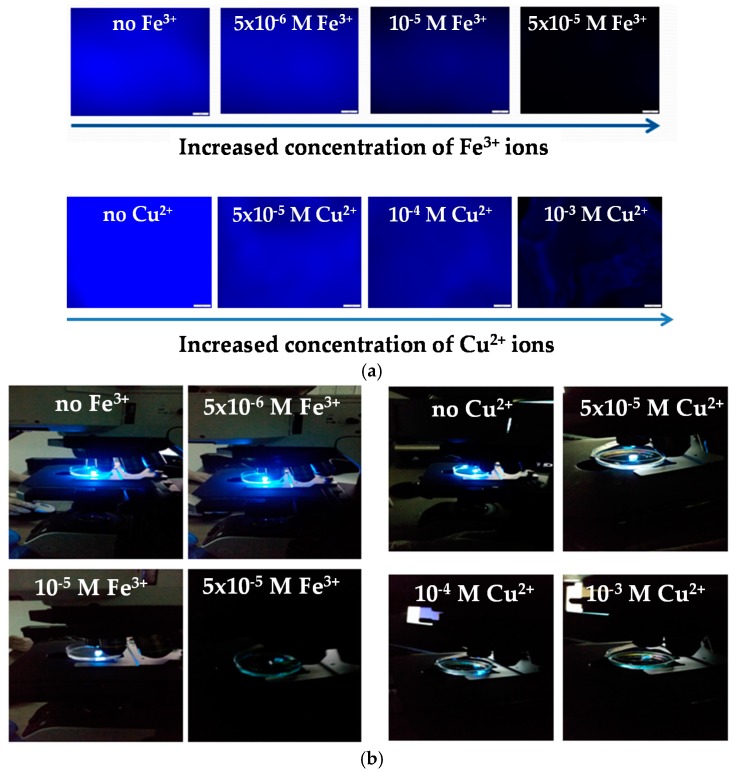
(**a**) Fluorescence images of the methanol-swollen BIEE/pDMAEMA/pAnMMA network immersed in pure methanol (control samples) and methanol solutions containing different Fe^3+^ and Cu^2+^ metal ion concentrations; (**b**) corresponding photographs. The scale bar in Figure 4a is 100 μm.

Figure 4a provides the fluorescence images corresponding to the BIEE/pDMAEMA/pAnMMA methanol-swollen network immersed in pure methanol (control samples) and methanol solutions containing different Fe^3+^ and Cu^2+^ metal ion concentrations. From the obtained fluorescence images, it can be clearly observed that the fluorescence intensity of the network is effectively quenched in the presence of both Fe^3+^ and Cu^2+^ metal ions, whereas quenching is more pronounced upon increasing the metal ion concentration as expected. During the analysis by means of fluorescence microscopy, photographs of the networks immersed in metal ion solutions of various concentrations were also taken in real time. As seen in Figure 4b, fluorescence quenching can be easily visualized, since upon immersion of the fluorescent network in the metal ion solutions its fluorescence efficiency reduces significantly.

**Figure 5:**
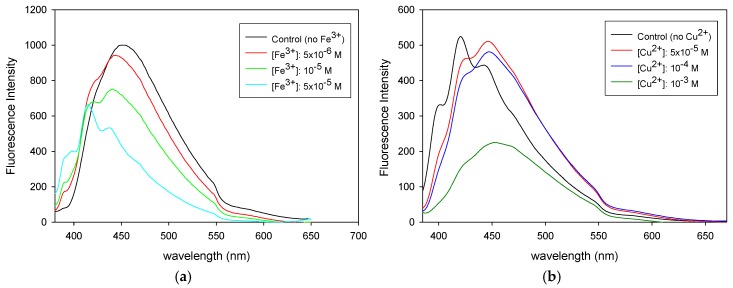
Fluorescence spectra of the methanol-swollen networks samples after being exposed to methanol solutions of various metal ion concentrations: (**a**) Fe^3+^ fluorescence monitoring; (**b**) Cu^2+^ monitoring.

Figure 5 presents the fluorescence spectra recorded for the methanol-swollen network samples after being immersed for 24 h in methanol solutions containing various concentrations of Fe^3+^ or Cu^2+^, followed by extensive washing to remove any unbound metal ions. As seen in the fluorescence spectra, a quenching effect is observed in both cases. This is attributed to the presence of unpaired d electrons in transition metal ions that can effectively quench the anthryl chromophore. The quenching phenomenon is further increased upon increasing the metal ion concentration, which is in agreement with our previous studies. Buruiana and co-workers reported on the different quenching mechanisms that may occur in anthracene-containing systems [74]. These include excimer or exciplex formation, metal–p complex, electron transfer, and energy transfer. In the case of Fe^3+^ possessing unpaired d electrons, the quenching mechanism may involve an energy transfer process from the singlet excited-state anthracene chromophores to the low-lying half-filled 3d orbitals of Fe^3+^ [75]. Besides the obvious decrease in the fluorescence intensity upon increasing the concentration of Fe^3+^, a blue shift is clearly observed in the fluorescence spectra (Figure 5a), whereas in the case of the Cu^2+^ the decrease in the fluorescence intensity is first preceded and then accompanied by a red shift (Figure 5b). According to Micheloni et al. [3], the coordination of metal ions may lead to an enhancement of the fluorescence emission (chelation-enhanced fluorescence effect, CHEF) or, as in the present study, to fluorescence quenching (chelation-enhancement quenching effect, CHEQ), whereas both effects may be coupled with a red or blue shift of the emission band.

According to the fluorescence spectroscopy data, in the case of the Cu^2+^ there is no obvious decrease in the fluorescence intensity until high Cu^2+^ concentrations (10^−3^ M) are reached, in contrast to the Fe^3+^ ions that act as effective quenchers for the anthracene fluorophores when present at much lower concentrations (~10^−5^ M). These results are in line with the fluorescence microscopy images provided in Figure 4.

**Figure 6:**
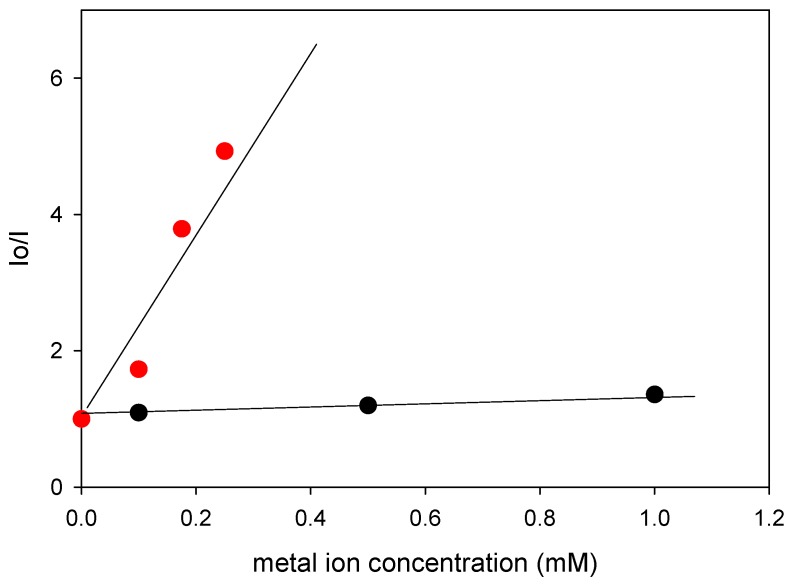
Stern–Volmer quenching plots of 9-anthracenemethanol in the presence of Fe^3+^ (red circles) and Cu^2+^ (black circles).

For further validation of the aforementioned results, control experiments were carried out in which 9-anthracenemethanol was used as the chemosensor in the monitoring of Fe^3+^ and Cu^2+^ in methanol. From the obtained data (Supplementary Materials, Figure S1, Table S1), the Stern–Volmer quenching plots were constructed (Figure 6), from which the Stern–Volmer quenching constants (Ksv) were determined from the Stern–Volmer equation, *I*_o_/*I* = 1 + *K*_sv_ × (*C*_ion_), to be (0.33 ± 0.04) and (16.5 ± 2.8) mM^−1^ for the Cu^2+^ and Fe^3+^ quenchers, respectively. *I*_o_ and *I* are the fluorescence intensities at ~410 nm in the absence and presence of the metal ions, respectively, and (*C*_ion_) is the concentration of the metal ion quencher. The obtained results are in very good agreement with the fluorescence spectroscopy data obtained in the case where the networks were used as chemosensors, thus confirming the higher sensitivity of these systems towards the fluorescence detection of Fe^3+^ over Cu^2+^.

The fact that in the control experiments using 9-anthracenemethanol as a chemosensor, which lacks metal ion-binding functionalities, no shift of the emission bands of the anthracene-containing fluorophore is observed in the presence of both metal ions (Supplementary Materials, Figure S1), strongly suggests that the semi-IPN network structure promotes the metal–ligand interactions via the metal-binding tertiary amino groups that are present on the pDMAEMA chains [58,59,60,61,62,63].

### 3.4. Metal Ion Desorption—Network Regeneration

**Figure 7:**
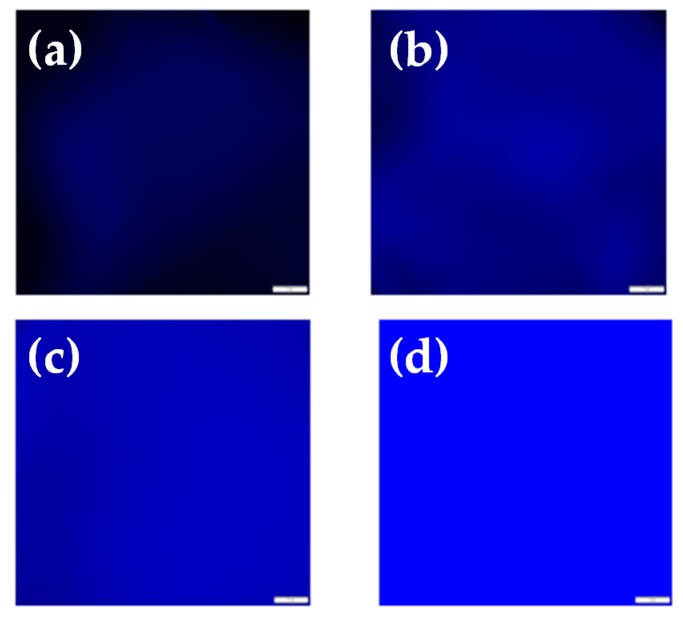
Fluorescence images analyzed using the CellSens software. Images (**a**,**b**) correspond to the Cu^2+^-loaded system equilibrated in 5 × 10^−3^ M Cu^2+^ methanol solution prior to and after immersion in the ethylenediaminetetraacetic acid (EDTA) aqueous solution, respectively. Images (**c**,**d**) correspond to the Cu^2+^-loaded system equilibrated in 10^−3^ M Cu^2+^ methanol solution prior to and after immersion in the EDTA aqueous solution, respectively. The scale bar in Figure 7 is 100 μm.

The desorption of Cu^2+^ ions from the networks, and thus their regeneration, was accomplished upon immersing two different samples of the Cu^2+^-loaded networks (pre-equilibrated in 10^−3^ and 5 × 10^−3^ M Cu^2+^ methanol solutions) in EDTA aqueous solutions. EDTA is a well-known chelating ligand that can bind onto various metal ions, including Cu^2+^, forming strong complexes. The metal ion desorption–regeneration process was qualitatively monitored in real time by fluorescence microscopy. As seen in the fluorescence images presented in Figure 7, an immediate increase in the fluorescence intensity of the networks was observed in both cases, qualitatively indicating desorption of the Cu^2+^ from the networks, thus resulting in the recovery of the networks’ fluorescence efficiency.

## 4. Conclusions

We have demonstrated a simple and versatile methodology that leads to the generation of 3D fluorescent semi-IPN amphiphilic polymer networks with controlled architectures, deriving from the well-defined molecular characteristics of their linear precursors. Fluorescence spectroscopy and microscopy demonstrated that these materials behave as strong blue emitters under UV irradiation in the presence of only 5 wt % of the pAnMMA linear chains that are interlaced between BIEE-crosslinked pDMAEMA segments. These systems were further evaluated as sorbents for the uptake and fluorescence monitoring of transition metal ions (Fe^3+^, Cu^2+^) in solution. The combination of the coordinating active part (pDMAEMA) with the fluorescent active component (pAnMMA) within a semi-IPN network architecture promotes a chelation-enhanced fluorescence quenching effect that is more pronounced in the case of Fe^3+^. Moreover, desorption of the Cu^2+^ from the networks could be realized upon immersion of the latter in an EDTA-containing solution, thus allowing their recyclability.

## Supplementary Materials

The following are available online at www.mdpi.com/2073-4360/8/12/411/s1, Table S1: Concentration of the metal ion quenchers (Cu^2+^, Fe^3+^) and *I*_o_/*I* data obtained by fluorescence spectroscopy when using 9-anthracenemethanol as a fluorophore; Figure S1: Fluorescence spectra of the 9-anthracenemethanol after being exposed to methanol solutions of various metal ion concentrations: Cu^2+^ fluorescence monitoring: left spectrum; Fe^3+^ monitoring: right spectrum.
