# Supplementary material for: Semi-Interpenetrating Polymer Networks with Predefined Architecture for Metal Ion Fluorescence Monitoring
Source: Polymers (Basel). 2016 Nov 29;8(12):411. doi: 10.3390/polym8120411 (PMC6431864; doi:10.3390/polym8120411)
Supplement: Supplementary file 1 [file polymers-08-00411-s001.pdf]

Supplementary Materials: Semi-Interpenetrating Polymer Networks with Pre-Defined Architecture for Metal Ion Fluorescence Monitoring

Kyriakos Christodoulou, Epameinondas Leontidis, Mariliz Achilleos, Christiana Polydorou and Theodora Krasia-Christoforou

Table S1. Concentration of the metal ion quenchers (Cu^{2+}, Fe^{3+}) and I_o/I data obtained by fluorescence spectroscopy when using 9-anthracenemethanol as a fluorophore.

**Cu^{2+}**		**Fe^{3+}**	
**Concentration (mM)**	**I_o/I (at λ = nm)**	**Concentration (mM)**	**I_o/I (at λ = nm)**
0	1.000	0	1.0000
0.1	1.093	0.1	1.7300
0.5	1.197	0.175	3.7900
1.0	1.359	0.25	4.9300

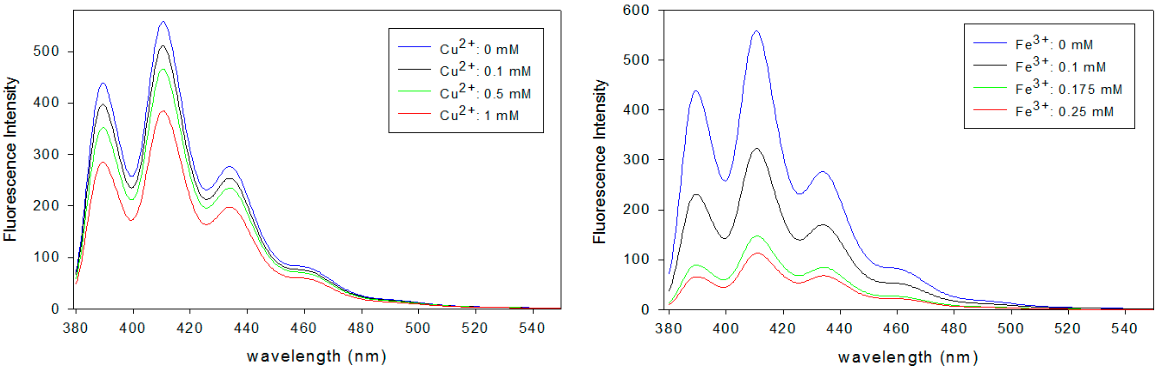

(**a**) (**b**)

Figure S1. Fluorescence spectra of the 9-anthracenemethanol after being exposed to methanol solutions of various metal ion concentrations: Cu^{2+} fluorescence monitoring (**a**); Fe^{3+} monitoring (**b**).
